# Does a self-referral counselling program reach doctors in need of help? A comparison with the general Norwegian doctor workforce

**DOI:** 10.1186/1471-2458-7-36

**Published:** 2007-03-16

**Authors:** Karin E Isaksson Rø, Tore Gude, Olaf G Aasland

**Affiliations:** 1 Department of Behavioural Sciences in Medicine, Institute of Basic Medical Sciences, Faculty of Medicine, University of Oslo, Norway Postbox 1111 Blindern, 0317 Oslo, Norway; 2 The Research Institute, Modum Bad, 3370 Vikersund, Norway; 3 The Research Institute, The Norwegian Medical Association, Postbox 1152 Sentrum, 0107 Oslo, Norway and Institute of Health Management and Health Economics, University of Oslo, Postbox 1111 Blindern, 0317 Oslo, Norway

## Abstract

**Background:**

Doctors have a relatively high degree of emotional distress, but seek help to a lesser degree and at a later stage than other academic groups. This can be deleterious for themselves and for their patients. Prevention programs have therefore been developed but it is unclear to what extent they reach doctors in need of help. This study describes doctors who participated in a self-referrral, easily accessible, stress relieving, counselling program in Norway, and compares them with a nationwide sample of Norwegian doctors.

**Methods:**

Two hundred and twenty seven (94%) of the doctors, 117 women and 110 men, who came to the resort centre Villa Sana, Modum, Norway, between August 2003 and July 2005, agreed to participate in the study. Socio-demographic data, reasons for and ways of help-seeking, sick-leave, symptoms of depression and anxiety, job stress and burnout were assessed by self-reporting questionnaires.

**Results:**

Forty-nine percent of the Sana doctors were emotionally exhausted (Maslach) compared with 25% of all Norwegian doctors. However, they did not differ on empathy and working capacity, the other two dimensions in Maslach's burnout inventory. Seventy-three percent of the Sana doctors could be in need of treatment for depression or anxiety based on their symptom distress scores, compared with 14% of men and 18% of women doctors in Norway. Twenty-one percent of the Sana doctors had a history of suicidal thoughts, including how to commit the act, as compared to 10% of Norwegian doctors in general.

**Conclusion:**

Sana doctors displayed a higher degree of emotional exhaustion, symptoms of depression and anxiety as well as job related stress, compared with all Norwegian doctors. This may indicate that the program at Villa Sana to a large extent reaches doctors in need of help. The counselling intervention can help doctors to evaluate their professional and private situation, and, when necessary, enhance motivation for seeking adequate treatment.

## Background

Emotional distress and impaired function among doctors can be deleterious for patients, as well as for the doctors themselves, their colleagues, other co-workers and families [1-3]. Doctors have a relatively high prevalence of depression, as well as higher suicide rates compared to other academic groups [4-8], and possibly also an increasing prevalence of burn-out [9]. Whereas these facts should lead to early helpseeking, doctors seem to seek help to a lesser degree, and later in the course of disease than do other groups [10,11]. Doctors do not easily adapt to the patient role and they also have problems being professional when treating a colleague [12,13]. Especially regarding mental health problems, doctors tend to be passive, embarrassed to seek help and worried about lack of confidentiality [2,14]. The term "hazardous heroes" has been used about doctors going to work with symptoms and problems for which they would have given their patients sick-leave [15].

Internationally it has been observed that doctors obtain treatment late in the course of emotional distress; many have been prompted by third parties such as insurance companies, police and review boards, which indicates that job performance has been impaired [16,17]. Treatment programs have with some success tried to lower the threshold for seeking treatment by letting the doctors use pseudonyms [16,18], offering treatment at one centralized national hospital in order to avoid the local environment [19], and by ensuring complete confidentiality, easy access and offices not identifiable with psychiatry [20,21].

The conditions described above have also led to the establishment of preventive programs. Primary and secondary preventive interventions for doctors have focused on developing appropriate stress management and coping skills [22-24]. Possible effects of such programs on emotional distress need further documentation. It is also important to explore the characteristics of the doctors recruited to such programs. Do the programs really reach doctors in need of help? To answer this, one needs to compare the help seeking group with the general doctor population. Few studies of this kind have been performed, and none in Norway.

In 1998, the Norwegian Medical Association (NMA) established a short-term counselling program for doctors, called Villa Sana. The declared aims for this effort were to enhance health and life quality, strengthen professional awareness and identity and prevent burnout. A prospective study of sociodemographic and clinical characteristics of doctors entering this program over a two-year period from August 2003 was initiated. The questionnaires were chosen in order to make the data comparable to those from a nationwide sample of Norwegian doctors. In this paper, we have raised the following research questions:

1. What characterizes doctors who seek help at Villa Sana with regard to gender, age and speciality compared to all Norwegian doctors?

2. a) How do doctors apply for help at Villa Sana, and what reasons do they give for seeking help?

b) To what degree did they seek help before coming to Villa Sana?

3. Do doctors seeking help at Villa Sana differ from Norwegian doctors concerning degree of job stress, burnout and symptoms of depression and anxiety?

### The Villa Sana program

The program offers two kinds of interventions for doctors or doctors with partners, who feel the need to reflect on their situation, related either to professional or private matters or both. One is a single day counselling session with one counsellor if the doctor comes alone, or two counsellors when there is also a partner or a spouse. The session lasts for 6–7 hours, aiming to give time and possibility to discuss the doctor's life situation and to suggest steps needed to handle the situation. This can include advice on seeking formal medical treatment including psychotherapy. The other type of intervention is a group-based, week-long course with boarding, dimensioned for eight individual doctors or four couples. There are daily lectures, group discussions and physical activity. One individual counselling session is offered during the week. Themes for the lectures are: possibilities and restraints in working life, the individual's resources and personality, concepts of identity, family relations, communication, team work and prevention of burnout. The group discussions are based on the participants' own experiences, providing the possibility to share these issues with colleagues.

The idea behind Villa Sana was that the program should be easily accessible. This includes making contact procedures as simple as possible, having readily available times for counselling appointments and having few restrictions as to reasons for contact. Doctors can contact Villa Sana directly on the phone, by post or by e-mail for an appointment. No referral is needed. All applicants are welcomed, except when need for immediate medical treatment is obvious, like serious risk for suicide or psychotic breakthrough. The program has been presented in the Norwegian Medical Journal and is announced twice a year in the same Journal. There are internet links to Villa Sana from the Norwegian Medical Association's web pages, as well as from Modum Bad's web pages. Members of the staff at Villa Sana are also invited to different physician meetings to inform about the program.

The Norwegian Medical Association covers all expenses, including travelling costs, making it possible for doctors from all parts of Norway to use Villa Sana. Since the program is defined as a preventive and not a clinical intervention, no medical records are kept.

## Methods

Of 242 eligible doctors, who participated in the Villa Sana programs between August 2003 and July 2005, 227 doctors (94%) gave their informed, written consent to participate in the study. Eighty-two percent (n = 187) came for a counselling session and 18% (n = 40) took part in the course program. The Sana doctors are compared with data from a survey of health, work conditions and life quality of Norwegian doctors from 1993 [25], comprising from 1009 – 6600 doctors depending on the object of inquiry.

### Specialities are grouped into

non-specialist, general practice (GP), internal medical specialities, surgical specialities, psychiatric specialities, public health and laboratory medicine [25].

### Ways of help-seeking

There are five dichotomous categories (0-no, 1-yes). If more than one category is marked yes, only one is chosen, ranking "referral" highest, "prompting by a colleague" second, "the doctor's own initiative" third, "coming because partner asked them to" fourth and "other reasons" fifth.

### Reason(s) for help-seeking

are defined as one or more of the following areas: health and life quality, exhaustion/burnout, professional identity (meaning identification with the role as a doctor) [26], work-related conditions, private relations and "other reasons" with an option for free text. Specified reasons under "other" were all, except one, possible to categorize under one of the first five areas – hence "other" is in the following omitted. Reasons are scored on a five-point scale (0- not decisive for help-seeking to 4-decisive for help-seeking). Scores 3 or 4 were considered "weighty reason".

### Job stress

A 26 items version of the original 43 items Cooper's job stress questionnaire [27], was used. Responses were given on a five-point scale with 1 = no stress and 5 = very much stress. This reduction was based partly on a Principal Component Analysis of data from a Norwegian student/doctor cohort from 1993 [28] yielding a 17-item version, and partly on the inclusion of nine other relevant items from the original instrument, amounting to 26 items (Cronbach's α = .90) with three subscales: emotional stress (10 items, α = .83), fear of litigation (6 items, α =.85), and social job-stress including time stress (10 items, α = .83).

### Burnout

Maslach's Burnout Inventory (MBI), with three sub-scales, was used – emotional exhaustion (10 items), reduced empathy (8 items), and reduced work capacity (7 items) [3,29]. Scores were given on a five-point scale (1 – does not fit, 5 – fits very well) evaluating the last two weeks of work. Reduced work capacity is presented with a reverse scale, so that a high value means low capacity. The dimensions are dichotomized in high and low, with cut-off >3 called case [3].

### General measure of depression and anxiety

Hopkin's symptom check list 5 (SCL-5) [30]was used. Answers were given on a five-point scale (1 – not at all, 5 – very much) evaluating the last two weeks. Studies on SCL-5 presented in literature, have used a four-point scale (1-not at all, 4-very much), with a cut-off of 1.67 for men and 1.75 for women indicating psychiatric case-ness/need for treatment [31]. Multiplying our cut-offs with 5/4 can give a case-estimate based on the five-point scale, yielding a cut-off of 2.09 for men and 2.19 for women. (Moum T, personal communication).

### Suicidal disposition

Measured by three of Paykel's five items [32]: "Have you ever had thoughts about taking your own life?", scored on a five-point scale (0 – never, 4 – very often), "Have you ever seriously considered taking your own life with plans on how to do so?" and " Have you ever attempted to commit suicide?"(both scored 0 – no and 1- yes).

### Sick leave

On sick-leave now (0-no, 1-on full-time sick-leave, 2- on part-time sick-leave, 3- full-time rehabilitation or disability leave, 4 – part-time rehabilitation or disability leave, 5 -on retirement).

### Present contact with GP

(0-no, 1-yes)

### Present contact with psychiatrist or psychologist

(0-no, 1-yes)

### Control samples

Due to the ten year gap between the 1993 survey and our study, we also investigated possible differences in comparable measurements with a nationwide medical student cohort followed from end of medical school (1993) throughout internship and residency (2003). The cohort is described by Tyssen et al [28], but the 10-year follow-up data from 2003 are not yet publicised (Røvik Jan Ole, personal communication). Controlled regarding gender and age, there were no significant differences on Hopkin's Symptom Check list (SCL5) nor on the dimension of emotional exhaustion (MBI) between the young doctor cohort in 2003 and all Norwegian doctors surveyed in 1993. Job stress showed a statistically significant, higher level in 2003, caused by the subdimension emotional stress: 1.97 (95% Confidence Interval [CI] 1.88–2.06) for 2003 versus 1.72 (95% CI 1.68–1.74) in 1993, p < 0.001. The two other job stress dimensions did not differ significantly. Suicidal measures were lower in 2003, but reported as last year's incidence, as opposed to life-time prevalence in 1993.

In addition, we have used general statistical data from the Norwegian Medical Association for 2004 and 2005, including doctors < 70 years (NMA web site).

### Statistics

Means, correlations, Students' t-test, Chi-square, ANCOVA, Logistic and Linear Regression Analysis were used, with the statistical program SPSS 13.0.

The study is approved by the Regional Ethical Committee for Ethical Research in the South of Norway and by the Data Inspectorate through the Norwegian Social Science Data Services.

## Results

### Gender, age and speciality

There were 117 (52%) women and 110 (48%) men in the Sana sample. For comparison on gender, age and speciality between the Sana-population and Norwegian doctors, see Table 1.

**Table 1:** Demographic data comparing Sana-doctors, Norwegian doctors 1993 and Norwegian doctors 2004/2005.

	**Sana-doctors **Mean (95%CI) Percentage (95% CI) n = 226	**Norwegian doctors 1993 **Mean (95%CI) Percentage (95% CI) n = 6602	Sign.	Norwegian doctors 2004/2005 * Mean Percentage n = 17500
**Age (years)**				
The whole population	46.9 (31.9–67.2)	42.5 (23.9– 60.7)	p < 0.001	45
Men	49.7 (32.0–67.3)	44.0 (25.4–62.6)	p < 0.001	
Women	44.3 (28.2– 60.4)	38.9 (21.5–56.3)	p < 0.001	
**Gender (%)**				
Men/Women	48 (42–55)/52 (45–58)	71 (70–72)/29 (28–30)	p < 0.001	64/36
**Specialities (%)**				
Non-specialist	30 (24–36)	42 (40–43)	p = 0.001	45
General practice	22 (17–28)	14 (13–15)	p = 0.001	13
Internal medical specialities	16 (11–21)	15 (14–16)	ns	15
Surgical specialities	15 (10–19)	14 (13–15)	ns	14
Psychiatric specialities	8 (5–12)	6 (5–7)	ns	6
Others (including social medicine and laboratory specialities)	8 (5–12)	9 (8–10)	ns	8

*Data from the Norwegian Medical Association. Doctors < 70 years.

The proportion of GPs was significantly higher in the Sana population (Odds Ratio (OR) = 1,67 95% Confidence Interval (CI) 1.20 to 2.32 p = 0.002), whereas other speciality differences between the two samples were not significant, when controlled for age and gender.

### Help-seeking

Fourty-five percent of the doctors came to Villa Sana on their own initiative, 37% reported that they were prompted by a colleague, 10% were referred, 6% came because they were asked by their partner and 2% came "for other reasons", without gender or age differences.

Weighty reasons for contacting Villa Sana are presented in Table 2. Most doctors state that health and life quality and exhaustion were weighty reasons for coming. More women than men stated exhaustion, and more men than women came for private reasons. Problems with professional identity were reported by 46% (95% CI 27–55) of those younger than 40 years, by 30% (95% CI 21–39) for those between 40–50 years and by 19% (95%CI 10–27) for those over 50 years of age. There is a significant difference between the youngest and the oldest group (p < 0.01).

**Table 2:** Reasons for seeking help.

**Weighty reasons for helpseeking **(>2 on a scale from 0–4)	**Total **% (95% CI) n = 224	**Men/Women **% (95% CI) n = 108/116	Sign.
Health and life quality	68 (62–74)	67 (58–76)/69 (60–77)	ns
Exhaustion/Burn-out	61 (55–68)	52 (42-–61)/69 (60–77)	P = 0.01
Private relations	56 (49–62)	65 (56–74)/48 (39–57)	p < 0.05
Work-related conditions	44 (38–51)	41 (31–50)/46 (37–55)	ns
Professional identity	28 (22–34)	25 (17–33)/31 (23–39)	ns

Proportions of doctors presenting each of the following reasons as a weighty reason for coming to Villa Sana. (Each doctor can present one or more reasons).

Among the Sana doctors two were recipients of 100% and four of 50% rehabilitation or disability benefits, and one doctor was retired. Among the remaining doctors (N = 219, one person not having answered) 41% (95% CI 33–47) were on sick-leave at the time of their first visit, with significant gender differences: 49% (95% CI 40–58) women (including 5 doctors on part-time sick-leave) versus 32% (95%CI 23–41) men (including 4 doctors on part-time sick-leave) p < 0.05. Age did not influence the proportion of doctors on sick-leave.

Thirty-nine percent had a present GP- contact, and 20% were in therapy with a psychiatrist or a psychologist when contacting Villa Sana.

### Distress assessments

The comparison between the Sana doctors and all Norwegian doctors regarding distress assessments showed that Sana doctors had significantly higher levels of emotional exhaustion (MBI), depression and anxiety (SCL5), all job stress dimensions and suicidal thoughts controlled for age and gender (Table 3). They did not have higher levels of reduced empathy or reduced work capacity (MBI).

**Table 3:** Comparison of burnout, SCL-5, job stress and suicidal thoughts between Sana-doctors and Norwegian doctors 1993.

	**Sana-doctors **Estimated Marginal Mean (95% CI of standard error)	**Norwegian doctors 1993 **Estimated Marginal Mean (95% CI of standard error)	Sign.
MBI – emotional exhaustion (1–5)	3.11 (3.00–3.22)	2.52 (2.46–2.57)	p < 0.001 ‡
MBI – reduced empathy (1–5)	1.96 (1.89–2.03)	1.92 (1.89–1.96)	ns † ‡
MBI – reduced capacity (1–5)	2.31 (2.25–2.37)	2.45 (2.42–2.48)	p < 0.001 ‡
SCL-5 (1–5)	2.91 (2.81–3.01)	1.55 (1.50–1.60)	p < 0.001
Job stress: Total (1–5)	2.51 (2.42–2.60)	2.02 (1.99–2.05)	p < 0.001 ‡
Job stress: Emotional (1–5)	2.19 (2.08–2.29)	1.70 (1.66–1.73)	p < 0.001 ‡
Job stress: Social (1–5)	2.88 (2.78–2.98)	2.28 (2.25–2.31)	p < 0.001 ‡
Job stress: Fear of litigation (1–5)	2.28 (2.17–2.39)	2.12 (2.08–2.15)	p < 0.01 ‡
Suicidal thoughts (0–4)	1.19 (1.08–1.30)	0.54 (0.49–0.60)	p < 0.001 ‡

Controlled for gender and age.

n for Sana = 222–227, n for Norwegian doctors 1993 1070 – for MBI and SCL-5, 1009 for suicidal thoughts, and 2497 for job stress.

For significant independent gender effect p < 0.01 = †

For significant independent age effect p < 0.01 = ‡

In the Sana group, 49% (95% CI 43 to 56) were defined as "cases" on the dimension of emotional exhaustion (MBI), compared to 25% (95% CI 22 to 28) among Norwegian doctors (p < 0.001).

Concerning SCL-5, cases with scores above cut-off and thus possibly in need of treatment were 73% (95% CI 64 to 81) among male Sana doctors versus 14% (95% CI 12–17) among Norwegian male doctors. Among women, 73% (95% CI 65–81) of the Sana doctors compared with 18% (95% CI 14–22) among Norwegian doctors scored above cut-off (p < 0.001 for both men and women).

Twenty-one percent (95% CI 15 to 26) of Sana doctors compared to 10% (95% CI 8 to 12) of all Norwegian doctors had seriously considered suicide, as well as having planned it (p < 0.001). Concerning attempted suicide, 2.6% (95% CI 0.6 to 4.7) of Sana-doctors compared to 1.6% (95% CI 0.8 to 2.3) of Norwegian doctors reported this (non significant difference).

## Discussion

One of the main findings in this study is the high level of distress among the Sana doctors compared with all Norwegian doctors, assessed with regard to emotional exhaustion, symptoms of depression and anxiety (SCL5 and suicidal thoughts and plans) and job stress parameters. Data indicate that 73% of the doctors coming to Villa Sana could be in need of treatment. The program thus reaches doctors who potentially are in serious need of help. Villa Sana being easily accessible, and the fact that the institution is not part of the ordinary medical services, probably facilitates help-seeking. The program seems to be a way of coming into contact with emotionally distressed doctors, where counselling can help them to evaluate their professional and private situation, in addition to enhancing their motivation for treatment, when necessary. Given the reluctance among doctors to ask for help [10,11], such motivation is probably important in a process of seeking adequate professional treatment.

Emotional exhaustion has, according to Falkum [3], been viewed by most authors as the primary dimension in a subsequent development of burnout. The apparent discrepancy in the Sana doctors between reporting high levels of emotional exhaustion, and at the same time not demonstrating reduced empathy or work capacity, is interesting, suggesting either that they take action in time to prevent burnout or stay working as "hazardious heroes", even with considerable distress, in order to fulfill their obligations and to maintain their self-esteem. Two recent studies have found a relationship between burnout dimensions and self-perceived sub-optimal patient care or medical mistakes, one of them suggesting a reciprocal cycle of the one triggering the other [33,34]. These studies, consonant with our findings, point to the importance of burnout prevention, both for the doctors' and their patients' well-being. More research concerning these relationships is needed [35].

Most of the doctors seeking help at Villa Sana present health issues, exhaustion and burnout as weighty reasons for coming. This is in line with the aims of the program, since it was established for potentially exhausted doctors. Among the younger doctors, problems with professional identity were found to be an important reason for help-seeking. This is consonant with a Finnish study, in which plans for changing career were associated with burnout especially among young doctors [36], as well as a study from the United States where career satisfaction showed a strong inverse relationship to burnout [33]. The early phases of the career, before speciality choices have been made, may be a period in which some doctors could benefit from counselling.

Although some studies indicate that female doctors have a higher prevalence of depression or minor psychiatric disease than male doctors [2,5,6,37], women doctors have not been found to have a higher degree of help-seeking than men [21,38]. The help-seeking studies however, refer mainly to younger doctors, whereas there are indications of increasing mental distress with age among women doctors, at least in the United Kingdom [6]. This might explain the higher proportion of women at Villa Sana. The issue of confidentiality could also be more important for women than for men [14], and Villa Sana provides a more confidential setting than ordinary psychiatric treatment.

The relative over-representation of GPs in the Sana group indicates that also Norwegian GPs experience more stress than other specialists, as found elsewhere [36,39,40].

Of the doctors who were already in therapy when contacting Villa Sana, many, according to our experience, wanted to discuss aspects of work. We have no registration of the intensity or type of therapy offered to these doctors and there might be several reasons for contacting Villa Sana while in therapy. One possibility is that some therapists and/or doctors view Villa Sana as a place of competence regarding problems related to doctors' work.

The comparative data used were collected ten years prior to our study, which is a limitation to interpreting the results. On the other hand, a more recent study in 2003 of Norwegian doctors, demonstrates that there is little difference between doctors in 1993 and 2003 on some important measures relevant to this study, indicating that the level of distress and mental health problems among Norwegian doctors has not changed dramatically over this period.

Another limitation is the self-reported information, including possible influence of distress symptoms on self-assessment of empathic aptitude and work capacity. The comparable data and studies referred to also use self-reporting forms, thus validating the comparison. There is, however, great need for a more objective evaluation of the relation between self-assessment and objective assessment as underlined in a recent review-article [41].

The major strength of this study is that it recruits a group of doctors seeking help at a counselling program, few studies of this kind having been done previously. The possibility to compare the help-seeking group with Norwegian doctors in general strengthens the design. The prospective aspect of the study enables us to follow the course of distress in the Sana doctors, one and three years after the short-term intervention they have been offered, and to document further help-seeking patterns.

## Conclusion

In comparison with all Norwegian doctors, this study shows high levels of emotional exhaustion, symptoms of depression and anxiety and job-related stress in doctors coming to an easily accessible, short term counselling program. In view of doctors' general reluctance to seek treatment, it is important to document that making a program easily accessible seems to be a way of reaching doctors in need of help. The counselling intervention can help doctors to evaluate their professional and private situation, and, when necessary, enhance motivation for treatment. Our prospective study will have the potential to document whether distress levels are reduced with time, and whether advice to seek treatment, given during the Sana intervention, is followed.

## Abbreviations

Norwegian Medical Association (NMA)

General Practioner (GP)

Hopkin's Symptom Check List with 5 items (SCL 5)

Maslach's Burnout Inventory (MBI)

Odds Ratio (OR)

Confidence Interval (CI)

Non significant (ns)

## Competing interests

The author(s) declare that they have no competing interests.

## Authors' contributions

KIR has had the main responsibility for this manuscript, with study design, development of the questionnaire used, data collection with analysis and interpretation and manuscript development.

TG has contributed to study design, development of the questionnaire used, to data analysis and interpretation and to manuscript development.

OA is responsible for data collection of the comparison sample, has participated in constructing the specific questionnaire used, and has contributed to manuscript revision.

All authors read and approved of the final manuscript.

## Pre-publication history

The pre-publication history for this paper can be accessed here:


